# Access to public health services and integral care for women during the puerperal gravid period period in Ceará, Brazil

**DOI:** 10.1186/s12913-019-4566-3

**Published:** 2019-11-20

**Authors:** Maria de Fátima Vasques Monteiro, Caio Parente Barbosa, Maria Auxiliadora Figueredo Vertamatti, Maria Nizete Alves Tavares, Amanda Cordeiro de Oliveira Carvalho, Ana Paula Agostinho Alencar

**Affiliations:** 1 Master in Nursing, Regional University of Cariri, Rua Coronel Antônio Luiz, 1161 - Pimenta, Crato, CE 63105-010 Brazil; 2 Faculty of Medicine of ABC, Santo André, São Paulo Brazil

**Keywords:** Women’s health, Child health, Health regulation and surveillance, Access to health services, Health completeness, Right to health

## Abstract

**Background:**

Over time, the Brazilian health system, a growing country, has been developing to ensure good accessibility to health goods and services. This development is focusing on the principle of universality of access and completeness of health care. In this context, we aimed to evaluate the completeness of care and universality of access for women in their pregnancy and puerperal period in Ceará, Brazil.

**Methods:**

A descriptive, cross-sectional study based on a quantitative approach, using information collected from the database of the regulation system of the state of Ceará and data from the Prenatal Monitoring System. The research population comprised of 1701 women who delivered a baby in an obstetric reference unit in the Health Macro-Region of Cariri, Ceará, Brazil from January to December 2015.

**Results:**

There was a high rate of cesarean delivery (49.7%) and a high waiting time for access to high-risk delivery (32.6%) and neonatal intensive care unit (72.9%). There was also a low percentage (41.1%) of pregnant women undergoing an adequate number of prenatal consultations, dental care (20%), educational activities (15%), visits to the maternity ward (0.1%), laboratory tests of the third trimester (29.2%) and puerperal consultation (37.9%).

**Conclusions:**

It was concluded that the Maternal and Child Health Policy, especially the Rede Cegonha, which is still under development, does not ensure access and completeness of care for women during the prenatal, delivery, and puerperal periods, thus violating their reproductive rights. The results of this study allow a critical analysis by the academia and health managers in search of strategies to improve the services of Rede Cegonha in Brazil.

## Background

The Brazilian Unified Health System (SUS), instituted by the Federal Constitution in 1988, is comprised of actions and services to promote health, protection and recovery of health implemented by federal entities, directly or indirectly, through the complementary participation of private initiative. The SUS is governed by some principles and guidelines that comprise of: universality, integrality, equity, regionalization, hierarchy, decentralization, single command and popular participation [1].

According to the Law of Health n^o^ 8.080 of 1990, which regulates SUS in Brazil, universality is the right of access to health services at all levels of care, guaranteed to all persons, regardless of gender, race, occupation, or other social or personal characteristics. Integrality of care is understood as articulated and continuous set of actions that involve preventive and curative services, individual and collective, required for each case at all levels of system’s complexity [2].

In pursuit of effectiveness of the principles of SUS, there was a creation of the Health Care Networks that originated from organizational networks connecting health care centers of lower and higher technological density, aimed at meeting the health needs of the population in the three levels of care integrating technical support, management, and logistics [3].

The Rede Cegonha, a Brazilian public health policy, is based on the Prenatal and Birth Humanisation Programme (Programa de Humanização no Pré-natal e Nascimento). With a focus on universality of access and efficient and effective completeness of care, both programmes aim to ensure improved access to and quality of prenatal, delivery and puerperium care, and child care [4].

In order to provide a complete care to women during pregnancy and the puerperal period, actions and services aimed at women’s care in pregnancy, delivery, and puerperium must be aligned with the criteria and parameters of SUS, such as prenatal consultation, dental care, educational activity, maternity visit, ultrasounds, laboratory examination, access to childbirth in a timely manner and home visit from a health professional during the puerperium. SUS estimates that 85% of pregnant women are at low-risk while 15% will face some degree of gestational risk and are therefore at high risk [5]. Health care provided for women should be intrinsically linked to the classification of pregnancy and delivery, aiming at a fair, integral, and individualised care.

Thus, the Brazilian Ministry of Health, through the Rede Cegonha, proposes the guarantee of prenatal consultations, laboratory and imaging support exams, visits to the maternity ward and delivery care, through actions of health promotion and prevention, educational activities and dental care, as standardised in the Prenatal and Birth Humanisation Programme [4].

However, in Brazil there is still inequity in access to health services by pregnant and parturient women and fragility of a complete care, humanisation, shelter, and bonding [6, 7]. It was concluded from some studies that some factors increase the difficulty to achieve integral care, such as: lack of communication and articulation among health establishments and sectors; discontinuation of health care and insufficient amount of human resources working in SUS [8].

The supply of health services has been insufficient for the demands of the population, pointing to the need for effective health systems and control of access through regulatory systems, in order to ensure the population accessible and comprehensive health care [3, 9, 10]. The supply should be increased to meet the demand. Recently, the Brazilian Ministry of Health approved criteria and parameters for the planning and programming of health actions and services, according to the number of pregnant women and children up to 2 years residing in the municipality or region, to be included in the prenatal, delivery and birth components of the Rede Cegonha [5].

Considering the universality of access and the completeness of the care as guiding principles in the SUS, the rights of the woman and duty of the State, as well as the effectiveness of public health policies focused on Health Care Systems (including the Maternal and Child System), the difficulty of the Brazilian health system - categorised as a universal health service - in ensuring integral care and providing equal opportunity of access to healthcare in a timely manner to health services and actions is a matter of concern.

The waiting time for a woman to receive healthcare plays an important role during puerperal pregnancy, on the health of pregnant women, mothers and newborns [11]. Research indicates that a waiting period of up to 6 h is considered clinically feasible to keep the prognosis unchanged [12, 13].

This problem is current and important for a country in which regional inequalities are so marked and political issues involving delivery and birth are subjects of great debate in the governmental and social scene, undergoing constant updates [14]. This study aims to evaluate the access and completeness of care for women during pregnancy and the puerperal period in a Macro-region of Ceará, Brazil. The Macro regions are formed by the grouping of health regions “a continuous geographic space constituted by groupings of bordering cities, delimited from cultural, economic and social identities and communication networks and shared transport infrastructure, in order to integrate the organization, the planning and execution of actions and services of health” [15].

Access was determined by the frequency of women who underwent prenatal consultations and puerperal visits, and the waiting time for a hospital vacancy. The completeness of care was assessed based on the frequency of procedures in the prenatal and puerperium components, according to the outcome variable delivery.

## Methods

This was a cross-sectional study based on data from the regulatory system of the state of Ceará and data from the Department of Informatics of SUS. The quantitative design made it possible to widely generalise the data collected, ensuring greater credibility and objectivity [16]. The population analysed consisted of 1701 women who delivered in an obstetric reference unit in the Health Macro-Region of Cariri, Ceará, Brazil, from January to December 2015.

We searched the regulation system for all deliveries, births, and waiting time of pregnant women and babies for obstetric beds and beds in the neonatal Intensive Care Unit (neonatal ICU). According to other studies in Brazil, the determinants of waiting time for care in SUS, related to pregnancy, delivery and puerperium, must be determined at local level, and are influenced by the State structure [11]. Thus, the parameters of the UNISUS System were used, which characterizes as possible waiting time for service, the intervals of < 2 h and 2 to 6 h and distinguishes the long waiting time as that occurs in the intervals 6 to 12 h and > 12 h. We used data from the Prenatal Monitoring System in order to evaluate access to promotion, prevention and treatment actions, which include educational activity, dental care, visits to the maternity ward, and puerperium consultation, among others.

Two spreadsheets were constructed for controlling data and ensuring data quality. One spreadsheet contained the variables of the Regulation System (UNISUS) and information on 100% of the deliveries performed in the obstetric reference unit, and the other contained information from the Information System for Monitoring and Evaluation of Prenatal, Delivery, Puerperium and Child (SISPRENATAL). In SISPRENATAL, we searched for the registration of women who delivered in the reference unit under study. The variables collected at UNISUS were date of request for a vacancy and date of hospitalisation, while the variables collected at SISPRENATAL were age, place of prenatal care, education level, marital status, access to prenatal care, dental care, educational activity, visits to the maternity ward, examination of images, laboratory examination, and puerperium consultation.

Stratified sampling was utilized and it was obtained by the calculation of a known population of Levin (1987) [17]. The data was processed by following these steps: data collection; tabulation of data with spreadsheet elaboration; construction of database. Statistical analysis was performed by Statistical Package for Social Sciences, version 13.0, Chicago, IL, USA for Windows. Descriptive analyzes were used for the absolute and relative frequencies and Chi-square test. For the inferential analysis, the data normality test (Kolmogorov-Smirnov) was performed, in which the non-normality of the data was identified (*p* > 0.05). Subsequently the U Mann Whitney test was performed to verify the difference between the groups. The significance was ≤0.05. All ethical and legal principles were met, and the research was approved by the Research Ethics Committee System of the Plataforma Brasil and the Regional University of Cariri under number 1,404,718.

## Results

Among the 1701 women who delivered in an obstetric reference unit in the Health Macro-Region of Cariri, Ceará, 50.3% had vaginal delivery and 49.7% had caesarean delivery. Among the pregnant women classified as high risk, most (66.4%) delivered by caesarean section (Table 1).

**Table 1:** Number of women attended to in the obstetric reference unit, by type of delivery and gestational risk classification, Ceará, Brazil, 2015. (*n* = 1701)

Variable	Normal Pregnancy		High-risk pregnancy		Total	
	n	%	n	%	n	%
Vaginal delivery	708	56.2	148	33.5	856	50.3
Caesarean delivery	551	43.8	294	66.5	845	49.7
Total	1259	74.0	442	26.0	1701	100

Source: Table prepared by the author based on data from UNISUS, Health Department of Ceará

With relation to the time of when women start giving birth to birth, there was a high frequency of waiting time in the range of 2 to 6 h for both normal (31.4%) and high-risk pregnancies (36.6%). When we analysed access to healthcare for newborns (*n* = 114), we found that 67.5% of babies waited > 12 h to access the neonatal ICU (Table 2).

**Table 2:** Number of women attended in an obstetric reference unit, by waiting time for delivery and birth, Ceará, Brazil, 2015 (*n* = 1815)

Variable	Delivery in Normal pregnancy		Delivery in high-risk pregnancy		Birth (neonatal ICU)		Total	
Waiting time	No.	%	No.	%	No.	%	No.	%
< 2 h	286	22.7	137	31.0	14	12.3	437	24.1
2 to 6 h	395	31.4	161	36.4	10	8.8	566	31.2
6 to 12 h	286	22.7	50	11.3	13	11.4	349	19.2
> 12 h	292	23.2	94	21.3	77	67.5	463	25.5
Total	1259	100.0	442	100.0	114	100.0	1815	100.0

Source: Table prepared by the author based on data from UNISUS, Department of Health of Ceará

Of the total 1701 women who delivered, all were registered in UNISUS records and 81.4% were registered in SISPRENATAL. We observed that the age group of 20–24 years was the most frequent (21%). When delivery in high-risk pregnancy was associated with age group, we observed that 12.4% of women who had deliveries in high-risk pregnancies were aged 15–19 years, when compared with delivery in normal pregnancy (3%). There a high percentage of the women (26%) were not registered in SISPRENATAL, which is justified by the non-registration of the women aged < 15 years. Most of the women completed high school (41.3%), and 62.9% of the women lived with their partners. However, of the 442 women who had deliveries in high-risk pregnancies, 9% lived with family members. It was found that normal and high-risk deliveries showed statistically significant differences depending on age group (Table 3).

**Table 3:** Number of women who delivered in conditions of normal pregnancy and high-risk pregnancy, by registration status, origin, social and demographic conditions, Ceará, Brazil, 2015. (*n* = 1701)

Characteristics	Normal		High Risk		Total	
	n	%	n	%	n	%
Registration						
Registered	1027	81.6	358	81,0	1385	81.4
Not registered	232	18.4	84	19.0	316	18.8
Age group						
15–19 years	38	03	55	12.4	93	05
20–24 years	249	19.8	105	23.8	354	21
25–29 years	238	18.9	90	20.4	328	19
30–34 years	197	15.6	58	13.1	255	15
> 34 years	181	14.4	56	12.7	237	14
No information	356	28.3	78	17.6	434	26
Education level						
Illiterate	11	0.9	3	0.7	14	0.8
Literate	162	12.9	64	14.5	226	13.3
Elementary school	92	7.3	50	11.3	142	8.3
High school	559	44.4	144	32.6	703	41.3
Did not complete high school	0	0	1	0.2	01	0.1
Higher education	62	4.9	23	5.2	85	5.0
Did not complete higher education	0	0	1	0.2	01	0.1
No information	373	29.6	156	35.3	529	31.3
Whom you live with						
Partner	821	65.2	248	56.1	1069	62.9
Family members	81	6.4	40	9.0	121	7.1
Alone	05	0.4	2	0.5	07	0.4
No information	352	28.0	152	34.4	504	29.6

Source: Table prepared by the author based on data from UNISUS, Department of Health of Ceará and SISPRENATAL, Ministry of Health

Table 4 shows that 41.1% of the women had more than six prenatal consultations. However, when analysed by gestational risk, women in normal pregnancy had more than six consultations more frequently (44.8%) than women in high-risk pregnancy (31.0%). Moreover, 30.5% of the women did not have information for monitoring prenatal delivery in high-risk pregnancy.

**Table 4:** Number of women delivering in a condition of normal pregnancy and in a condition of high-risk pregnancy, by prenatal and puerperium variables, Ceará, Brazil, 2015. (*n* = 1701)

Variables	Delivery in normal pregnancy		Delivery in high-risk pregnancy		Total	
	n	%	n	%	n	%
Number of consultations						
1–5 consultations	464	36.9	170	38.5	634	37.3
> 6 consultations	563	44.8	137	31.0	700	41.1
No information	232	18.3	135	30.5	367	21.6
Dental care						
Performed	221	17.6	126	28.5	347	20.4
Not performed	805	63.9	185	41.9	990	58.2
No information	233	18.5	131	29.6	364	21.4
Educational activity						
Performed	136	10.8	118	26.7	254	15.0
Not performed	890	70.7	193	43.7	1083	63.7
No information	233	18.5	131	29.6	364	21.3
Visit to the maternity ward						
Yes	02	0.2	0	0	02	0.1
No	1024	81.3	311	70.4	1335	78.5
No information	233	18.5	131	29.6	364	21.4
Imaging examinations–obstetric ultrasound						
Performed	845	67.1	231	52.3	1076	63.5
Not performed	181	14.4	80	18.1	261	15.3
No information	233	18.5	131	29.6	364	21.4
Laboratory tests						
1st trimester	534	42.4	232	52.5	766	45.0
1st and 3rd trimesters	442	35.1	54	12.0	496	29.2
Not performed	50	4.0	09	2.0	59	3.5
No information	233	18.5	147	33.3	380	22.3
Puerperium consultation						
Yes	454	36.1	190	43.0	644	37.9
No	572	45.4	121	27.4	693	40.7
No information	233	18.5	131	29.6	364	21.4

Source: Table prepared by the author based on data from UNISUS, Secretariat of Health of Ceará and SISPRENATAL, Ministry of Health

It can be observed that 20.4% of pregnant women received dental care, 15% received some educational activity, and 0.1% visited the maternity ward. Moreover, women delivering in a condition of high-risk pregnancy showed a higher percentage (29.6%) of dental care and educational activity (26.7%), when compared with women delivering in normal conditions (Table 4).

The analysis of the information presented in Table 4 also showed that 74.2% of pregnant women underwent laboratory tests (Hemogram, Blood Type, Fasting Glycemia, Urine Summary, and other tests through the blood fluid that detects diseases like HIV, Syphilis among others), while only 63.5% of them underwent imaging tests (obstetrical ultrasonography). We observed that in the first trimester of pregnancy, 45% of the women had access to laboratory tests for ABO/Rh, haemoglobin/haematocrit, and human immunodeficiency virus, in addition to the first urine, blood glucose, and venereal disease research laboratory tests. Conversely, when they were compared to women who underwent tests in the 1st and 3rd trimesters, the percentage dropped significantly, with most of them, 29.2%, not having access to the second urine, glycaemia, and venereal disease research laboratory test. Still, with regard to laboratory tests, we found that 12% were women with high-risk pregnancies and 35.1% of those with normal pregnancies did not undergo tests in both trimesters. Finally, the percentage of women who did not have puerperium consultations (40.7%) was higher than those who had (37.9%). There were statistically significant differences to the access of healthcare (*p* = 0.000) and within completeness by comparing the number of visits (*p* = 0.001), dental care (*p* = 0.000), educational activities 0.002) and laboratory tests (*p* = 0.000) among women who presented the usual risk and high risk in deliveries. Maternity visits and puerperium consultation did not present statistically significant differences in women with normal delivery and high-risk delivery.

## Discussion

With regard to the frequency of births, a high rate of caesarean deliveries was observed. These data are alarming when compared to the rate of 15% recommended by the international medical community and the World Health Organization (WHO). Similar results were found in national and international studies [18–20].

In Brazil, considering that caesarean delivery has been growing significantly, the topic is a cause for concern. The World Health Organization characterises caesarean delivery as an epidemic, since it is high in both developed and underdeveloped countries.

The outcome of caesarean section during high-risk pregnancy found in this study is similar to the findings obtained in other states in the northeast and southeast Brazil [20, 21]. It is emphasised that caesarean delivery, when performed following medical and scientific indications, contributes to the reduction of maternal and perinatal mortality, especially in high-risk pregnancies [19, 22].

The planning parameters of health actions and services for pregnancy, delivery, and puerperium care in Brazil classifies that 15% of women progress to high-risk pregnancy deliveries [5]. However, our study obtained a higher rate, which raises prerogatives for the establishment of new parameters in Brazil according to the specificities of each region.

When identifying the waiting time for regulation of delivery in normal pregnancy or of high risk, a higher frequency was found within 2 to 6 h of waiting for access to the delivery unit. Conversely, a higher frequency of waiting longer than 12 h was observed for access to beds in the neonatal ICU. Both findings demonstrate the fragility of the principle of universality of timely access to the services of the Rede Cegonha in the scenario under study. Corroborating the fragility of health services, they point to inadequate care for the reproductive needs of Brazilian women.

The inadequate provision of neonatal ICU beds compromises the guarantee of timely access [23, 24], proving that the expansion of neonatal beds is necessary. It is emphasised that even in the richest regions of the country it is difficult to obtain vacancies for high-complexity services in the SUS despite the prioritisation of care in the most emerging cases from the regulation of access [25]. The results show that the mother and child pair still face administrative barriers that cause loss of time and commitment in the continuity and completeness of care.

With regard to the sociodemographic data of this study, most patients were aged between 20 and 34 years. However, we observed a higher frequency of delivery in high-risk pregnancy in women aged less than 19 years. Most of the women had completed high school and lived with their partners.

With regard to the frequency of prenatal procedures, we observed that most of the women had access to the consultation, however not all performed the six consultations as recommended [26]. Other studies show that the minimum number of six prenatal visits is not adequately performed in several states of Brazil [27, 28].

There is a significant difference in the number of prenatal consultations carried out in the public and private system, resulting in the difficulty of access to actions that promote and control complications arising during pregnancy and delivery [29]. The results found—and the studies mentioned corroborate—that there is a discontinuity and fragmentation of care offered by the SUS to women during delivery and the puerperal period.

Most of the women did not have access to health promotion and prevention actions in the prenatal period, (including dental care, educational actions and visits to the maternity ward), which demonstrates the fragility of access by pregnant women to complementary prenatal care actions recommended by the Rede Cegonha.

Educational practises allow the empowerment of women in coping with the daily situations of pregnancy and enable co-responsibility of the family through increased acceptance of the physical and emotional changes of women during this period [30, 31]. However, this study points to weaknesses in health education actions for women linked to prenatal care in the Basic Health Units.

Other studies have obtained similar results [18, 32], highlighting that prenatal care in the SUS basic health system is permeated by the fragility of health care associated with a low resolution of primary care, a fact that compromises the principle of universality and completeness of care.

Another deficient factor in the SUS is the communication between the primary and secondary health care services, reflected by the lack of dialogue between the care centres, due to the unavailability of data systems to keep electronic medical records, distancing pregnant women from the services that provide delivery care and, consequently, resulting in women delivering in maternity wards that they had not visited previously [32].

Still, with regard to the prenatal component of the Rede Cegonha, it is of paramount importance to have access to quality imaging and laboratory tests, performed promptly, in order to strengthen the actions of prevention and early detection of diseases that might affect the health of pregnant women and foetuses [33]. However, in this study, there was a reduced percentage of women registered for all these exams, pointing to a deficiency in the offer of diagnostic support services in pregnancy and puerperal period.

The lack of prenatal care in the SUS care system has contributed to undesirable outcomes such as premature births and low birth weight, triggering an increase in maternal and infant mortality [34].

The absence of access to puerperium consultation compromises the completeness of care. In this study, the low percentage of women who had a puerperal consultation corroborates the findings of another study, highlighting the deficiency in home visits by the Family Health Strategy [18].

The underfunding of the federal government for the health of Brazilians brings to the SUS a shortage of beds and limitation of specialised services, making the balance between supply and demand unfeasible. Moreover, the repressed demand for procedures of medium and high complexity contribute to the restriction of access, making it unfeasible to meet health needs and consequently compromising the implementation of comprehensive care.

## Conclusions

It was verified that despite the implementation of Rede Cegonha in the region, the actions and health services are fragmented and disarticulated, without communication between the health care services. The services should be timely and continuous to meet the real need of women during the gestational period. However, in the current context, the discontinuation of access of health actions and services in the three levels of assistance, disqualifying the concept of integrality of maternal and child care are seen. Consequently, the Maternal and Child Health Policy in the Cariri Macro-region in Ceará, especially the Rede Cegonha, is failing to ensure the right to health, access, and full care to women during the prenatal, delivery, and puerperal periods.

Therefore, we suggest expanding the study to other health macro-regions by expanding the sample size since there are regional inequalities in the different population dimensions of the Brazilian territory.

## Data Availability

The statistical data used and analysed in this study are available in the UNISUS and SISPRENATAL systems. The interview data generated and analysed during the present study are not publicly available to protect the anonymity of study participants.

## Abbreviations

Neonatal ICU: Neonatal Intensive Care Unit
SISPRENATAL: Information System for the Monitoring and Evaluation of Prenatal, Delivery, Puerperium, and Child
SUS: Unified Health System
UNISUS: Regulation System

## References

[1] Brasil (1988). Constituição (1988). Constituição da República Federativa do Brasil.

[2] Brasil (1990). Lei n° 8.080, de 19 de setembro de 1990. Dispõe sobre as condições para a promoção, proteção e recuperação da saúde, a organização e o funcionamento dos serviços correspondentes e dá outras providências.

[3] Mendes EV (2011). As redes de atenção à saúde.

[4] Ministério da Saúde (2011). Portaria n° 1.459, de 24 de junho de 2011. Institui no âmbito do Sistema Único de Saúde a Rede Cegonha.

[5] Ministério da Saúde. Portaria n° 1.631, de 01 de outubro de 2015. Aprova critérios e parâmetros para o planejamento e programação de ações e serviços de saúde no âmbito do SUS: Diário Oficial da União; 2011. http://bvsms.saude.gov.br/bvs/saudelegis/gm/2015/prt1631_01_10_2015.html. Viewed 27 Feb 2019

[6] Sousa LMO, Araújo EM, Miranda JGV (2017). Caracterização do acesso à assistência ao parto normal na Bahia, Brasil, a partir da teoria dos grafos. Cad Saúde Pública.

[7] Cabral FB, Hirt LM, Sand ICPV (2013). Atendimento pré-natal na ótica de puérperas: da medicalização à fragmentação do cuidado. Rev Esc Enferm USP.

[8] Silva RVGO, Ramos FRS (2010). Integralidade em saúde: revisão da literatura. Cienc Cuid Saude.

[9] Santos ACGE, Tanaka OY (2011). Financiamento, gasto e oferta de serviços de saúde em grandes centros urbanos no estado de São Paulo. Cien Saúde Colet.

[10] Patel P, Das M, Das U (2018). The perceptions, health-seeking behaviours and access of scheduled Caste women to maternal health services in Bihar, India. Reprod Health Matters.

[11] Marinho A, Cardoso SC (2007). Um estudo multinível sobre as filas para internações relacionadas com a gravidez, o parto e o puerpério no SUS. Econ Aplic.

[12] Goldwasser RS, Lobo MS, Arruda EF, Angelo SA, Ribeiro EC, Silva JR (2018). Planning and understanding the intensive care network in the state of Rio de Janeiro (RJ), Brazil: a complex societal problem. Rev Bras Ter Intensiva.

[13] Chalfin DB, Trzeciak S, Likourezos A, Baumann BM, Dellinger RP (2007). Impact of delayed transfer of critically ill patients from the emergency department to the intensive care unit. Crit Care Med.

[14] Dias MAB (2011). Humanização do parto: política pública, comportamento organizacional e ethos profissional. Cad Saúde Pública.

[15] Ministério da Saúde (BR) (2011). Secretaria de Gestão Estratégica e Participativa. Decreto n° 7.508, de 28 de junho de 2011: regulamentação da Lei n° 8.080/90.

[16] Leopardi MT (2002). Metodologia da pesquisa na saúde.

[17] Costa CSC, Vila VSC, Rodrigues FM, Martins CA, Pinho LMO (2013). Característica do atendimento pré-natal na rede básica de saúde. Rev Eletr Enf.

[18] Schantz C, Sim KL, Petit V, Rany H, Goyet S (2016). Factors associated with caesarean sections in Phnom Penh, Cambodia. Reprod Health Matters.

[19] Barros AJD, Santos IS, Matijasevich A, Domigues MR, Silveria M, Barros FC (2011). Patterns of deliveries in a Brazilian birth cohort: almost universal cesarean sections for the better-off. Rev Saúde Pública.

[20] Leal RC, Santos CNC, Lima MJV, Moura AOP, Pedrosa AO, Costa ACM (2017). Complicações materno-perinatais em gestação de alto risco. Rev Enferm UFPE on line.

[21] Viana RC, Novaes MRCG, Calderon IMP (2011). A Mortalidade Materna – uma abordagem atualizada. Comun Cienc Saúde.

[22] Angelo SA, Arruda EF, Goldwasser R, Lobo MSC, Salles A, Silva JRL (2017). Demand forecast and optimal planning of Intensive Care Unit (ICU) capacity. Pesqui Oper Rio de Janeiro.

[23] Tajra FS, Pontes JS, Carvalho FHC (2016). Diálogos sobre regionalização, redes e regulação em saúde. Rev Enferm UFPI.

[24] Santos AM, Giovanella L (2016). Gestão de cuidados integral: estudo de casos em região de saúde da Bahia, Brasil. Cad Saúde Pública.

[25] Ministério da Saúde. Portaria n° 1.026, de 29 de maio de 2013. Institui as diretrizes para a organização da Atenção à saúde na gestação de alto risco: Diário Oficial da União; 2013. http://bvsms.saude.gov.br/bvs/saudelegis/gm/2013/prt1020_29_05_2013.html. Viewed 27 Feb 2019

[26] Parada CMGL (2008). Avaliação da assistência pré-natal e puerperal desenvolvidas em região do interior do Estado de São Paulo em 2005. Rev Bras Saude Mater Infant.

[27] Coutinho T, Monteiro MF, Sayd JD, Teixeira MT, Coutinho CM, Coutinho LM (2010). Monitoring the prenatal care process among users of the Unified Health Care System in a city of the Brazilian Southeast. Rev Bras Ginecol Obstet.

[28] Paris GF, Pelloso SM, Martins PM (2013). Qualidade da assistência pré-natal nos serviços públicos e privados. Rev Bras Ginecol Obstet.

[29] Facchini LA, Tomasi E, Dilélio AS (2018). Quality of primary health care in Brazil: advances, challenges and perspectives. Debate de Saúde.

[30] Souza VB, Silva JS, Barros MC, Freitas PSP (2014). Tecnologias leves na saúde como pontencializadores para qualidade da assistência às gestantes. Rev Enferm UFPE on line.

[31] Vasconcelos MFF, Nicolotti CA, Silva JF, Pereira SMLR (2016). Entre políticas (EPS – Educação Permanente em Saúde e PNH – Política Nacional de Humanização): por um modelo de formar no/para o Sistema Único de Saúde (SUS). Inferface.

[32] Anversa ETR, Bastos GAN, Nunes LN, Dal Pizzol TS (2012). Qualidade do processo da assistência pré-natal: Unidade Básica de Saúde e Unidades de Estratégias de Saúde da Família em um munícipio no Sul do Brasil. Cad Saúde Pública.

[33] Martinelli KJ, Neto ETS, Gama SGN, Oliveira AE (2014). Adequação do processo da assistência pré-natal segundo os critérios do Programa de Humanização do Pré-natal e Nascimento e Rede Cegonha. Rev Bras Ginecol Obstet.

[34] Levin J. Estatística aplicada a ciências humanas. 2ª ed. São Paulo: Ed. Harba;1987.

